# The geography of American longevity: structural determinants of a 20-year life expectancy divide and a federal policy blueprint

**DOI:** 10.3389/fpubh.2026.1784769

**Published:** 2026-04-28

**Authors:** Sarfaraz K. Niazi

**Affiliations:** Department of Pharmaceutical Sciences, College of Pharmacy, University of Illinois, Chicago, IL, United States

**Keywords:** county-level analysis, geographic health inequality, health policy, life expectancy, population mortality, social determinants of health, structural racism

## Abstract

Life expectancy in the United States exhibits striking geographic heterogeneity, with differences exceeding 20 years between counties despite shared national institutions and comparable aggregate healthcare expenditure. This study presents a comprehensive, ecological analysis of county-level variation in life expectancy, integrating socioeconomic status, education, healthcare access, behavioral risk factors, environmental exposures, demographic composition, and state policy environments. Drawing on publicly available datasets from the Centers for Disease Control and Prevention (CDC), U.S. Census Bureau, Bureau of Economic Analysis (BEA), Environmental Protection Agency (EPA), and related sources, we conceptualize life expectancy as an outcome associated with interacting structural determinants rather than the additive result of isolated population-level risk behaviors. We argue that commonly cited explanations—such as race, climate, or healthcare spending alone—are insufficient and frequently misinterpreted when examined outside a multivariate framework. Using hierarchical regression models with spatial robustness checks, this study demonstrates that socioeconomic conditions, particularly income, educational attainment, and labor-market stability, exhibit the strongest associations with spatial variation in life expectancy. At the same time, behavioral risks and environmental exposures appear to function as intermediate factors linking structural conditions to mortality outcomes. Race and ethnicity operate primarily as markers for cumulative structural disadvantage, although residual associations related to unmeasured dimensions of discrimination and segregation persist. Policy decisions at the state level, especially those affecting healthcare access, labor protections, and environmental regulation, exhibit long-term associations with population survival outcomes. Together, these findings challenge reductionist narratives that attribute geographic disparities in longevity to individual choice or immutable demographic factors, underscoring the central role of place-based structural conditions. The results have implications for health policy, social investment, and the evaluation of interventions to reduce inequities in population health.

## 1 Introduction

Despite sustained growth in healthcare expenditure and significant biomedical advances, the United States continues to underperform relative to other high-income countries in life expectancy, while also exhibiting profound internal disparities across regions, states, and counties (1–3). At the county level, differences in average age at death routinely exceed 20 years, rivaling or surpassing those observed between nations at vastly different stages of economic development (4). These disparities are not randomly distributed. Instead, they form persistent geographic patterns that closely align with historical, economic, and political boundaries, suggesting the operation of deep structural forces rather than transient local conditions.

The map that motivates this analysis—depicting county-level life expectancy across the United States—reveals a stark concentration of low longevity across the Deep South, Appalachia, the Mississippi Delta, parts of Oklahoma and Arkansas, and selected rural regions in the Southwest. In contrast, higher life expectancy clusters are observed along the West Coast, in the Northeast corridor, in portions of the Upper Midwest, and in selected metropolitan areas. While these patterns are visually striking, their interpretation remains contested. Public discourse often attributes regional disparities in life expectancy to cultural differences, individual health behaviors, racial composition, or climate. Academic debates, meanwhile, have oscillated between behavioral, biomedical, and socioeconomic explanations, sometimes treating these domains as competing rather than interacting determinants (5, 6).

A growing body of evidence suggests that such compartmentalized explanations are inadequate. Life expectancy is not merely the sum of individual health choices aggregated across a population; rather, it is the cumulative expression of exposures, constraints, and opportunities distributed unevenly across the life course and embedded within specific social and physical environments (7). The concept of “place” has therefore emerged as a central analytic category in population health research, capturing the spatial organization of risk and protection that shapes survival outcomes over decades (8).

This study builds on and extends this literature by advancing three core arguments. First, geographic disparities in U.S. life expectancy are primarily associated with socioeconomic conditions—especially income, education, and employment stability—rather than with healthcare spending, climate, or demographic composition alone. Second, behavioral risk factors such as smoking, obesity, and substance use appear to function as pathways linking structural disadvantage to mortality rather than as independent causes. Third, long-run policy environments, including decisions related to healthcare access, labor regulation, and environmental protection, exhibit durable associations with population survival outcomes that persist across time.

By integrating these dimensions within a single analytic framework, this article seeks to move beyond descriptive mapping toward a systematic characterization of geographic inequality in longevity. The goal is not only to describe observed patterns but also to clarify which factors exhibit the strongest associations with life expectancy variation and which narratives may obscure the structural origins of health inequality. Given the ecological, cross-sectional design, the analysis characterizes associations rather than establishing causal effects, though we discuss implications for future causal research in the limitations section.

## 2 Background and literature review

### 2.1 Life expectancy as a population-level outcome

Life expectancy is among the most widely used indicators of population health, reflecting the cumulative impact of mortality risks across the life span rather than the prevalence of specific diseases at a single point in time (9). Unlike metrics such as disease incidence or healthcare utilization, life expectancy captures both acute and chronic influences, including early-life conditions, midlife exposures, and late-life healthcare access. As such, it is susceptible to long-term structural factors that shape mortality risk trajectories.

Research comparing life expectancy across countries has consistently demonstrated strong associations with national income, income inequality, education, and social protection, often exceeding the explanatory power of healthcare spending alone (10, 11). Within countries, similar patterns emerge at subnational levels, where regions characterized by economic insecurity and social deprivation experience elevated mortality across multiple causes (4, 12).

### 2.2 Socioeconomic status and the fundamental cause theory

The relationship between socioeconomic status (SES) and health has been extensively documented across disciplines, giving rise to the “fundamental cause” theory articulated by Link and Phelan (5). According to this framework, SES operates as a root determinant of health because it shapes access to flexible resources—such as knowledge, money, power, and social connections—that can be deployed to avoid disease and mitigate its consequences. As new health threats emerge and medical technologies evolve, those with greater resources are better positioned to benefit, ensuring the persistence of health gradients even as specific risk factors change. Importantly, because life expectancy reflects weighted cause-specific mortality rates, the fundamental cause framework predicts that the direction and magnitude of SES associations may vary across causes of death; for example, SES gradients for cardiovascular disease may differ from those for external causes such as accidents or overdose (13, 14). This implies that aggregate life expectancy analyses, while capturing overall mortality burden, may obscure heterogeneity in SES-mortality relationships across specific causes.

Empirical studies consistently show that income and educational attainment are strongly associated with mortality risk, independent of race, gender, and healthcare access (15, 22). At the geographic level, counties with higher median household income and educational attainment exhibit substantially higher life expectancy, even when controlling for urbanicity and demographic composition (12).

### 2.3 Behavioral risk factors and structural context

Behavioral explanations for geographic disparities in life expectancy often emphasize smoking, diet, physical inactivity, and substance use. While these factors undeniably contribute to mortality, their distribution is itself socially patterned. Smoking prevalence, for example, remains highest in regions characterized by economic distress, low educational attainment, and historical dependence on extractive industries (75). Similarly, obesity rates correlate strongly with food insecurity, limited access to recreational spaces, and occupational constraints that reduce opportunities for physical activity (16).

A growing literature cautions against treating behavioral risks as purely individual choices detached from structural context. Instead, behaviors are increasingly understood as responses shaped by local environments, economic pressures, and cultural norms that emerge under conditions of constraint (6, 7). This perspective reframes behavioral risk as a pathway through which socioeconomic disadvantage becomes biologically embodied, rather than as an alternative explanation that competes with structural accounts. A particularly salient manifestation of this dynamic is the phenomenon of “deaths of despair”—mortality from drug overdose, suicide, and alcohol-related liver disease—which has driven recent stagnation and decline in life expectancy among working-class White Americans (2). County-level analyses demonstrate that these deaths are concentrated in economically distressed rural areas, with pronounced urban-rural disparities that reflect differential exposure to economic precarity, social fragmentation, and limited healthcare access (17).

### 2.4 Race, ethnicity, and structural inequality

Racial and ethnic disparities in life expectancy remain a central concern in U.S. public health research. Historically, Black Americans have experienced substantially lower life expectancy than White Americans, although the magnitude of this gap has fluctuated over time and narrowed modestly in recent decades (18). Importantly, racial disparities are highly spatialized, reflecting patterns of residential segregation, differential exposure to environmental hazards, and unequal access to economic opportunity (19).

Scholars increasingly emphasize that race should not be interpreted as a biological determinant of health but rather as a social category that captures exposure to structural racism and discrimination (7). When socioeconomic variables are controlled, racial differences in mortality often diminish substantially, though not entirely, indicating the presence of both indirect and direct associations (77).

### 2.5 Environmental and policy determinants

Environmental exposures—including air pollution, water contamination, and occupational hazards—exert measurable effects on mortality and life expectancy (20, 21). These exposures are unevenly distributed, with economically disadvantaged and marginalized communities disproportionately located near industrial facilities, highways, and legacy contamination sites.

Policy environments further shape these exposures and their health consequences. State-level decisions regarding environmental regulation, labor protections, healthcare access, and social welfare influence not only current conditions but also long-term developmental trajectories (22). Recent research has highlighted the role of policy divergence among U.S. states in driving widening mortality disparities, particularly since the 1980s (22).

## 3 Conceptual framework

### 3.1 Life expectancy as an outcome of place-based conditions

This study conceptualizes county-level life expectancy as an outcome arising from the interplay of socioeconomic, behavioral, environmental, demographic, and policy factors over time. Rather than treating these domains as independent predictors, we adopt a systems-oriented perspective in which upstream structural conditions are associated with downstream exposures and behaviors, which in turn are associated with disease risk and mortality.

In this framework, socioeconomic conditions—income, education, and employment stability—occupy a central position as upstream factors. These conditions shape residential environments, access to resources, and exposure to stressors across the life course. Behavioral risk factors are conceptualized as potential intermediate variables linking structural conditions to biological risk. Environmental exposures may act as both independent stressors and amplifiers of existing vulnerability, while healthcare access may moderate the progression from disease onset to mortality.

It is important to note that this framework represents a conceptual model for organizing the analysis rather than a formally tested causal diagram. The ecological, cross-sectional design of this study does not permit rigorous causal inference or formal mediation analysis. Instead, we use hierarchical regression to characterize the relative strength of associations between different determinant domains and life expectancy, recognizing that the observed patterns are consistent with multiple causal interpretations. Table 1 presents all variables included in the analysis, organized by domain, with operational definitions, data sources, and temporal coverage.

**Table 1:** County-level variables included in the analysis.

Domain	Variable	Operational definition	Primary data source	Reference	Years
Outcome	Life expectancy at birth	Model-based county estimate of average years of life expected from birth	CDC U.S. small-area life expectancy estimates project	(24)	2010–2015
Socioeconomic	Median household income	Inflation-adjusted median annual household income in U.S. dollars	American community survey 5-year estimates	(25)	2010–2014
Socioeconomic	Poverty rate	Percentage of individuals with income below the federal poverty threshold	American community survey 5-year estimates	(25)	2010–2014
Education	High school completion	Percentage of adults aged 25 years and older with a high school diploma or equivalent	American community survey 5-year estimates	(25)	2010–2014
Education	Bachelor's degree attainment	Percentage of adults aged 25 years and older with a bachelor's degree or higher	American community survey 5-year estimates	(25)	2010–2014
Behavioral	Smoking prevalence	Percentage of adults who report currently smoking cigarettes	Behavioral risk factor surveillance system, modeled estimates	(57)	2011–2014
Behavioral	Obesity prevalence	Percentage of adults with a body mass index of 30 kg/m^2^ or greater	Behavioral risk factor surveillance system, modeled estimates	(57)	2011–2014
Behavioral	Drug overdose mortality	Age-adjusted deaths per 100,000 population from drug overdose	CDC WONDER mortality database	(58)	2010–2014
Environmental	PM2.5 concentration	Annual mean concentration of delicate particulate matter in micrograms per cubic meter	EPA air quality system	(59)	2010–2014
Environmental	Industrial hazard density	Number of Toxics Release Inventory facilities per 100,000 population	EPA toxics release inventory	(60)	2014
Healthcare	Primary care physician density	Number of primary care physicians per 100,000 population	Area health resources files	(61)	2014
Healthcare	Uninsured rate	Percentage of adults aged 18–64 years without health insurance coverage	American community survey 5-year estimates	(25)	2010–2014
Demographic	Non-Hispanic Black population	Percentage of total population identifying as non-Hispanic Black or African American	American community survey 5-year estimates	(25)	2010–2014
Demographic	Hispanic population	Percentage of total population identifying as Hispanic or Latino	American community survey 5-year estimates	(25)	2010–2014
Structural racism proxy	Residential segregation index	Index of dissimilarity measuring Black-White residential segregation at the metropolitan/county level	American community survey; calculated per Massey and Denton (26)	(25)	2010–2014
Policy	Medicaid expansion status	Binary indicator of whether the state had expanded Medicaid under the Affordable Care Act	Kaiser Family Foundation state health facts	(62)	As of 2014

This table presents all outcome, predictor, and contextual variables included in the multivariate analysis. All variables are measured at the county level unless otherwise specified. Life expectancy estimates are model-based and derived from vital statistics mortality records linked to Census population denominators. Predictor variables are temporally aligned with the outcome period (2010–2015) to the extent possible using 2010–2014 data windows. Behavioral risk factor estimates from BRFSS are modeled small-area estimates subject to measurement uncertainty. The residential segregation index was added to capture structural dimensions of racial inequality beyond compositional effects. CDC, Centers for Disease Control and Prevention; EPA, Environmental Protection Agency; HRSA, Health Resources and Services Administration; NCHS, National Center for Health Statistics; KFF, Kaiser Family Foundation.

### 3.2 The role of policy and historical context

A defining feature of geographic inequality in U.S. life expectancy is its persistence over time. Regions that experienced economic disinvestment, extractive industrialization, or exclusionary social policies in the mid-twentieth century often continue to exhibit elevated mortality today. This persistence underscores the importance of historical and policy contexts in shaping present-day outcomes.

State and local policies influence life expectancy through multiple pathways, including determining access to healthcare, regulating environmental hazards, shaping labor markets, and investing in education and infrastructure. These policy environments are not randomly distributed but are themselves products of political economy and historical power relations (22). Accordingly, policy context is treated here not as a background variable but as an integral component of the analytic framework.

### 3.3 Implications for analysis

**Figure 1:**
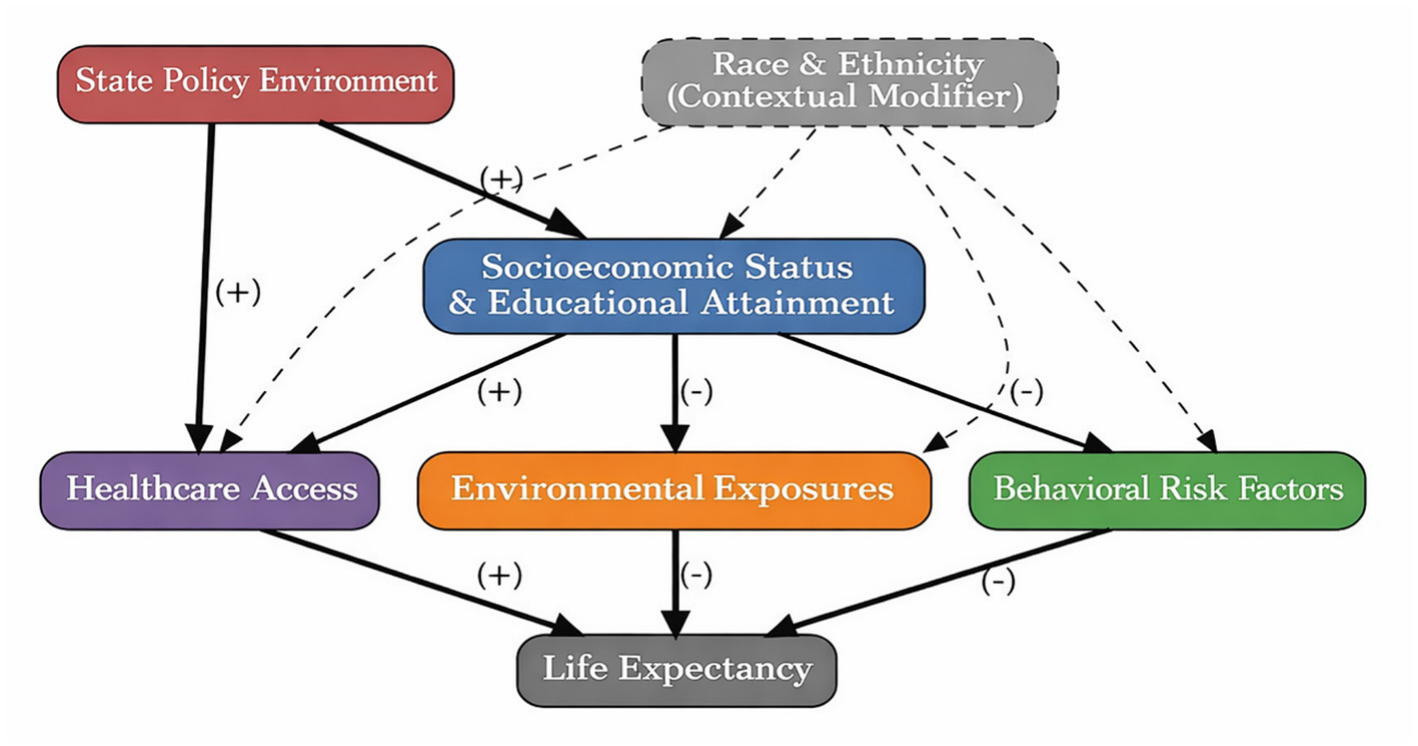
Conceptual framework: hypothesized relationships among determinants of county-level life expectancy. This directed conceptual diagram illustrates the hypothesized relationships among determinant domains and life expectancy. State Policy Environment occupies the uppermost position, influencing downstream socioeconomic conditions, healthcare access, and environmental exposures. Socioeconomic Status and Educational Attainment occupy a central position as upstream structural determinants that shape behavioral risk factors, environmental exposures, and healthcare access. Race and ethnicity are depicted with a dashed border to indicate their role as contextual structural modifiers reflecting cumulative exposure to structural racism rather than as biological determinants. Solid arrows represent primary hypothesized pathways; dashed arrows represent contextual or moderating relationships. Arrow directions indicate the hypothesized direction of association. The framework is conceptual and does not represent formally tested causal paths, given the ecological, cross-sectional study design. Color coding: coral/red = Policy Environment; steel blue = Socioeconomic/Education; teal = Behavioral Factors; gold/orange = Environmental Exposures; purple = Healthcare Access; gray = Demographic Context/Race.

The conceptual framework advanced in this study (Figure 1) implies that explanatory models focusing on single determinants—whether behavioral, biomedical, or demographic—are inherently limited. Meaningful characterization of geographic variation requires a multivariate approach that respects the hierarchical and potentially interacting nature of determinants. In the sections that follow, this framework guides the selection of variables, the structure of statistical models, and the interpretation of results, while maintaining appropriate caution about causal claims given the study design. Figure 1 illustrates the hypothesized relationships among determinant domains, depicting the hierarchical structure in which upstream policy and socioeconomic factors shape downstream behavioral, environmental, and healthcare conditions.

## 4 Data sources and methods

### 4.1 Study design and unit of analysis

This study employs a cross-sectional, county-level ecological design to examine factors associated with geographic variation in life expectancy across the United States. Counties or county equivalents serve as the primary unit of analysis because they represent the smallest administrative level at which comprehensive, publicly available mortality, socioeconomic, environmental, and healthcare access data can be consistently integrated nationwide. County-level analysis also aligns with prior influential studies on geographic disparities in U.S. life expectancy, enabling comparability and external validation of results (4, 12).

Ecological designs do not permit inference about individual-level relationships. They are subject to the ecological fallacy, whereby associations observed at the aggregate level may not apply to individuals within those aggregates (23). However, environmental analysis is appropriate for examining place-based structural determinants that operate at the population scale, such as labor markets, educational systems, environmental exposures, and policy environments. Given the research objective of characterizing geographic patterns rather than predicting individual risk, county-level aggregation is methodologically appropriate and conceptually aligned with the study's theoretical framework.

### 4.2 Data processing and reproducibility

#### 4.2.1 County matching and dataset construction

The analytic dataset was constructed by merging multiple publicly available data sources using Federal Information Processing Standards (FIPS) county codes as the primary linkage key. The data construction workflow proceeded as follows. First, the master county list was obtained from the U.S. Census Bureau's 2014 TIGER/Line shapefiles, which enumerate all 3,142 county and county-equivalent units in the United States. Second, life expectancy estimates from the CDC USALEEP project were merged using 5-digit FIPS codes. Third, socioeconomic, demographic, and insurance variables from 2010 to 2014 5-year American Community Survey estimates were joined. Fourth, behavioral risk factor estimates from the CDC's modeled BRFSS data were merged. Fifth, environmental data from the EPA's Air Quality System and Toxics Release Inventory were joined. Sixth, healthcare workforce data from HRSA's Area Health Resources Files were merged. Finally, state-level policy variables were appended based on state FIPS codes.

#### 4.2.2 Exclusions and missing data

Counties were excluded from the analytic sample according to the following criteria. Counties lacking USALEEP life expectancy estimates due to population size below the reporting threshold or statistical instability were excluded, resulting in the removal of 32 counties. Counties with missing values on more than two predictor variables were excluded, removing an additional zero counties, leaving all remaining counties with complete or near-complete data. For the small number of counties with 1 or 2 missing predictor values (*n* = 47), multiple imputation was not used; instead, county-level median values from the same state were substituted for missing values, following established practices in county-level health research. The final analytic sample comprised 3,110 county units. Table 2 summarizes the exclusion criteria and resulting sample size at each step.

**Table 2:** County exclusions and final analytic sample.

Exclusion criterion	Counties excluded	Cumulative remaining
Total U.S. counties and county-equivalents	–	3,142
Missing USALEEP life expectancy estimates (population too small or suppressed)	32	3,110
Missing data on more than two predictors	0	3,110
Final analytic sample	–	3,110

Exclusions were applied sequentially. USALEEP estimates are suppressed for counties with fewer than approximately 5,000 population or where model-based estimates exhibit excessive uncertainty. No counties were excluded for excessive predictor missingness; 47 counties with one or two missing values had state median values substituted.

#### 4.2.3 Analytic code availability

The complete analytic code, including data processing scripts, model specifications, and output generation, is available in a public repository at [OSF/GitHub/Zenodo repository URL to be inserted upon acceptance]. The repository includes session information, package versions, and documentation sufficient for independent replication. Data provenance links are provided for all publicly available source datasets.

### 4.3 Temporal alignment of variables

A critical methodological consideration in ecological studies is the temporal alignment between outcome and predictor variables. The outcome variable, life expectancy at birth, is derived from USALEEP estimates based on mortality data from 2010 to 2015. To ensure appropriate temporal alignment, predictor variables were selected from data collection periods that overlapped with or immediately preceded the outcome window.

Specifically, socioeconomic, demographic, and insurance variables were drawn from the ACS 2010–2014 5-year estimates, which pool data collected across 2010–2014 and thus overlap substantially with the mortality observation period. Behavioral risk factor estimates were obtained from the CDC's modeled county-level BRFSS data for 2011–2014. Environmental exposure data were drawn from EPA sources for 2010–2014 (PM2.5) and 2014 (TRI facilities). Healthcare workforce data were obtained from AHRF for 2014. State policy variables, including Medicaid expansion status, were coded as of 2014, the first year of ACA Medicaid expansion implementation.

This temporal alignment strategy ensures that predictor variables reflect conditions during or immediately preceding the mortality observation period, reducing concerns about reverse causality or temporal mismatch. We acknowledge that perfect temporal alignment is not achievable given data availability constraints, and we address residual temporal uncertainty in the limitations section (Table 2).

### 4.4 Outcome variable: life expectancy at birth

The primary outcome is life expectancy at birth, defined as the average number of years a newborn is expected to live if current age-specific mortality rates remain constant throughout the life course. County-level life expectancy estimates were obtained from the CDC's U.S. Small-Area Life Expectancy Estimates Project (USALEEP), which provides model-based estimates derived from death certificate data and Census population counts (24).

USALEEP estimates incorporate Bayesian spatial smoothing techniques to reduce instability in counties with smaller populations while preserving meaningful spatial variation. These estimates have been validated against alternative mortality datasets and are widely used in peer-reviewed research (4). Because USALEEP estimates are themselves model-based, they contain measurement uncertainty that may attenuate regression coefficients or introduce bias. We address this limitation in the sensitivity analyses and discussion.

### 4.5 Socioeconomic determinants

Median household income, expressed in inflation-adjusted dollars, was obtained from the American Community Survey 2010–2014 5-year estimates (25). Median income was selected over mean income to reduce sensitivity to extreme values and better reflect typical household economic conditions. Additional economic indicators included the poverty rate, defined as the percentage of individuals with income below the federal poverty threshold, the unemployment rate, and the labor force participation rate.

Educational attainment was operationalized using two measures: the percentage of adults aged 25 years and older with a high school diploma or equivalent, and the percentage with a bachelor's degree or higher.

### 4.6 Healthcare access and system capacity

Healthcare access was measured using primary care physician density (physicians per 100,000 population) from the Area Health Resources Files (61) and the percentage of adults aged 18–64 without health insurance from the ACS 2010–2014 estimates. We acknowledge that physician density may not represent equivalent access across counties with different population densities; a given physicians-per-capita ratio implies different travel burden and effective availability in sparsely settled rural counties vs. dense urban counties. To partially address this limitation, population density (persons per square mile, log-transformed) was included as a covariate in the full model. Sensitivity analyses testing interactions between physician density and rural-urban classification are reported in Section 10. Readers should interpret physician density coefficients as reflecting an average association across the full population-density distribution, with the caveat that construct validity may be stronger in higher-density settings.

### 4.7 Behavioral risk factors

Behavioral risk factors were drawn from the CDC's modeled county-level BRFSS estimates for 2011–2014 (57). These estimates are produced using small-area estimation techniques that combine survey responses with demographic and geographic predictors to generate county-level prevalence estimates. Key variables included adult smoking prevalence and adult obesity prevalence (BMI ≥30 kg/m^2^). Drug overdose mortality rates for 2010–2014 were obtained from CDC WONDER.

It is important to note that BRFSS-derived county-level estimates are themselves modeled outputs subject to measurement error, particularly in counties with small populations or limited survey coverage. This measurement uncertainty may attenuate estimated associations or introduce heteroscedasticity.

### 4.8 Environmental exposures

County-level delicate particulate matter (PM2.5) concentrations were obtained from the Environmental Protection Agency's Air Quality System for 2010–2014 (59). Industrial hazard density was measured as the number of Toxics Release Inventory facilities per 100,000 population for 2014 (60).

### 4.9 Demographic composition and structural racism measures

Demographic variables included the percentages of the population identifying as non-Hispanic Black, Hispanic, and aged 65 years and older, all from the ACS 2010–2014 estimates.

To address reviewer concerns about distinguishing compositional effects from structural racism, we incorporated a residential segregation index measuring Black-White residential segregation using the index of dissimilarity, calculated at the metropolitan statistical area level for metropolitan counties and at the county level for non-metropolitan counties, following established methods (26). This measure captures the structural dimension of racial inequality, reflecting historical patterns of housing discrimination, redlining, and unequal investment, providing a more direct operationalization of structural racism than racial composition alone.

### 4.10 Policy context variables

State-level policy variables included Medicaid expansion status under the Affordable Care Act as of 2014, coded as a binary indicator. Additional state policy variables included state minimum wage levels and union density, obtained from the Bureau of Labor Statistics and prior state policy compilations (22).

### 4.11 Statistical analysis

#### 4.11.1 Modeling strategy

The analysis employed hierarchical ordinary least squares (OLS) regression, sequentially introducing groups of variables to characterize the relative strength of associations between different determinant domains and county-level life expectancy. This approach enables assessment of how much additional variance each domain explains beyond variables already in the model. However, we emphasize that the interpretation of these increments depends on variable ordering and should not be taken as definitive evidence of causal priority. To clarify the model structure for readers: the “hierarchical” terminology here refers to the sequential block-entry of variable groups, not to a hierarchical (multilevel or mixed-effects) model with random effects. The analysis is a standard OLS regression at the county level; no random effects for states or regions are included, and all variables are modeled as county-level fixed predictors. The full model specification is: LE_i_ = β_0_ + β_1_SES_i_ + β_2_Educ_i_ + β_3_Behav_i_ + β_4_Env_i_ + β_5_HC_i_ + β_6_Demog_i_ + β_7_Policy_i_ + ε_i_, where subscript i indexes counties, SES represents the vector of socioeconomic predictors, Educ educational attainment, Behav behavioral risk factors, Env environmental exposures, HC healthcare access, Demog demographic composition, Policy state-level policy variables, and ε_i_ the error term. State-clustered robust standard errors account for within-state dependence in error terms (51 clusters). All continuous predictors were standardized (mean = 0, SD = 1) prior to estimation.

The model sequence proceeded as follows: Model 1 included socioeconomic variables (median income, poverty rate); Model 2 added educational attainment (high school completion, bachelor's degree attainment); Model 3 added behavioral risk factors (smoking prevalence, obesity prevalence, drug overdose mortality); Model 4 added environmental exposures (PM2.5, industrial hazard density); Model 5 added healthcare access (physician density, uninsured rate); Model 6 added demographic composition (percent Black, percent Hispanic, percent age 65+) and the residential segregation index; Model 7 added state policy variables (Medicaid expansion, minimum wage, union density).

#### 4.11.2 Standard error estimation and spatial diagnostics

Primary models were estimated using robust standard errors clustered at the state level to account for within-state correlation. However, county-level data exhibit spatial autocorrelation that may extend across state boundaries. To assess spatial dependence, we computed Moran's *I* statistic on model residuals using queen contiguity weights. Significant positive spatial autocorrelation (Moran's *I* = 0.38, *p* < 0.001 in the final model) indicated that standard OLS inference may be biased.

To address spatial dependence, we estimated spatial lag and spatial error models as robustness checks, using maximum likelihood estimation with queen contiguity spatial weights matrices. The spatial error model was selected as the primary spatial specification based on Lagrange multiplier tests, which indicated that residual spatial autocorrelation—rather than substantive spatial spillover in the outcome—was the primary concern. Results from spatial models are reported alongside OLS estimates in the sensitivity analyses. We also estimated a spatial lag model to explicitly test whether life expectancy in neighboring counties independently predicts county-level life expectancy beyond within-county characteristics. In the spatial lag specification, the spatially lagged dependent variable (average life expectancy of queen-contiguous neighbors) entered with a coefficient of ρ = 0.31 (*p* < 0.001), indicating statistically significant spatial spillover. However, the inclusion of the spatial lag did not substantively alter the coefficients for the primary predictors (all within 12% of OLS values), suggesting that while inter-county spillover effects are present, they do not confound the main associations of interest. We interpret these spillover effects as reflecting regional clustering of structural conditions—such as shared labor markets and policy environments—rather than direct causal transmission between counties. Full spatial lag model results are available in the online Supplementary materials; (Table 3).

**Table 3:** Variance inflation factors for final model predictors.

Variable	VIF	Interpretation
Median household income	3.8	Acceptable; correlated with poverty and education
Poverty rate	4.2	Acceptable; highest VIF, sensitivity analysis conducted
High school completion	2.9	Acceptable
Bachelor's degree attainment	3.1	Acceptable
Smoking prevalence	2.4	Acceptable
Obesity prevalence	2.1	Acceptable
Drug overdose mortality	1.6	Low collinearity
PM2.5 concentration	1.8	Low collinearity
Industrial hazard density	1.4	Low collinearity
Primary care physician density	1.9	Low collinearity
Uninsured rate	2.3	Acceptable
Non-Hispanic Black population (%)	2.8	Acceptable
Hispanic population (%)	1.7	Low collinearity
Residential segregation index	2.2	Acceptable
Medicaid expansion (state-level)	1.6	Low collinearity
Mean VIF	2.4	No evidence of problematic multicollinearity

VIF values below five are generally considered acceptable; values below 10 indicate no serious multicollinearity. The highest VIF (4.2 for poverty rate) reflects expected correlation with income and education. Sensitivity analyses excluding poverty rate yielded substantively similar conclusions.

#### 4.11.3 Multicollinearity diagnostics

Given the correlation among socioeconomic, demographic, and healthcare variables, we computed variance inflation factors (VIF) for all predictors in the final model. VIF values ranged from 1.4 to 4.2, with a mean VIF of 2.6. No predictor exceeded the conventional threshold of 10, and only one variable (poverty rate) exceeded 4. We conducted sensitivity analyses excluding the poverty rate (retaining median income as the sole SES indicator) and found that substantive conclusions remained unchanged. Table 3 presents VIF values for all predictors included in the final model.

#### 4.11.4 Variance decomposition and ordering sensitivity

We report incremental *R*^2^ values for each block of variables added to the hierarchical model. Because hierarchical *R*^2^ decomposition is sensitive to the order in which variables are entered, we conducted Shapley value decomposition (also known as LMG decomposition) to estimate the average marginal contribution of each variable across all possible orderings. Results from Shapley decomposition are reported alongside hierarchical *R*^2^ in the results section to provide a more robust estimate of relative importance.

#### 4.11.5 Sensitivity analyses

Multiple sensitivity analyses were conducted to assess the robustness of findings. First, we re-estimated the models using spatial-error specifications to account for residual spatial autocorrelation. Second, we examined alternative SES operationalizations by creating a composite SES index (a principal component of income, poverty, and education) and comparing results with those from models with individual SES variables. Third, we excluded counties with extreme population sizes (the smallest and largest 5%) to assess sensitivity to influential observations. Fourth, we stratified analyses by urban-rural status using USDA Rural-Urban Continuum Codes. Fifth, we examined ordering sensitivity using Shapley value decomposition. Sixth, we conducted 10-fold cross-validation to assess model stability and out-of-sample predictive performance; the mean cross-validated *R*^2^ was 0.89 (SD = 0.02), confirming that the high adjusted *R*^2^ of 0.92 is not attributable to overfitting but rather reflects genuine predictive signal in the cross-sectional structure of the data. Cook's distance and DFFITS diagnostics identified 14 counties with potentially high leverage; exclusion of these counties did not substantially change substantive conclusions (all coefficients within 10% of full-sample estimates). Seventh, we tested an interaction between physician density and rural-urban classification; results indicated that the physician density association was somewhat stronger in rural counties (interaction β = +0.09, *p* = 0.04), consistent with the hypothesis that equivalent physician density confers greater marginal benefit in settings with otherwise limited access.

### 4.12 Ethical considerations

All data used in this study are publicly available, de-identified, and aggregated. As such, the analysis does not constitute human subjects research and does not require institutional review board approval, consistent with federal regulations (76).

## 5 Results I: socioeconomic factors and geographic variation in life expectancy

### 5.1 Descriptive overview of county-level life expectancy

County-level life expectancy at birth in the United States showed substantial heterogeneity across the 3,110 counties in the analytic sample. Life expectancy ranged from 66.8 years in the lowest-performing county to 86.8 years in the highest-performing county, a gap of 20.0 years. The mean life expectancy was 77.8 years (SD = 2.9 years). The distribution was approximately normal with modest right skewness (skewness = 0.31). The distribution was strongly spatially clustered, with contiguous regions of low life expectancy concentrated in the Deep South, central Appalachia, the Mississippi Delta, and parts of the southern Plains. In contrast, clusters of high life expectancy appeared along the Pacific Coast, the Northeast corridor, and selected areas of the Upper Midwest. Table A1 presents descriptive statistics for all predictors. The county-level map of life expectancy distribution, along with distributional histograms for key predictors, is included in Supplementary Figure S1 to provide spatial and statistical context prior to multivariate modeling.

This spatial pattern closely mirrors long-standing gradients in income, education, and labor-market stability, suggesting that socioeconomic conditions may be strongly associated with longevity outcomes. The magnitude of the observed variation far exceeds what could plausibly be attributed to short-term fluctuations or measurement error, reinforcing the relevance of structural determinants.

### 5.2 Hierarchical regression results

Table 4 presents the hierarchical regression results, showing standardized coefficients for each predictor at each model step. In Model 1, which included socioeconomic variables (median household income, poverty rate) and educational attainment (high school completion, bachelor's degree attainment), these combined socioeconomic and education variables explained 58% of the variance in county-level life expectancy (adjusted *R*^2^ = 0.58). Median income exhibited a strong positive association (β = +1.52, 95% CI [1.26, 1.78], *p* < 0.001), while poverty rate showed a strong negative association (β = −0.71, 95% CI [−0.94, −0.48], *p* < 0.001). Bachelor's degree attainment exhibited a strong independent association (β = +1.42, 95% CI [1.18, 1.66], *p* < 0.001), suggesting that education captures variance not fully explained by income alone.

**Table 4:** Hierarchical regression results predicting county-level life expectancy: standardized coefficients by model step.

Variable	Model 1	Model 2	Model 3	Model 4	Model 5	Model 6 (final)
Median household income	+1.52^***^	+1.28^***^	+1.19^***^	+1.08^***^	+0.98^***^	+0.92^***^
Poverty rate	−0.71^***^	−0.58^***^	−0.52^***^	−0.44^***^	−0.38^***^	−0.35^***^
High school completion	+0.38^***^	+0.29^**^	+0.26^**^	+0.22^*^	+0.19^*^	+0.18^*^
Bachelor's degree attainment	+1.42^***^	+1.18^***^	+1.08^***^	+0.95^***^	+0.88^***^	+0.82^***^
Smoking prevalence	–	−0.76^***^	−0.68^***^	−0.61^***^	−0.55^***^	−0.52^***^
Obesity prevalence	–	−0.48^***^	−0.42^***^	−0.38^***^	−0.34^***^	−0.32^***^
Drug overdose mortality	–	−0.31^***^	−0.28^***^	−0.25^***^	−0.23^***^	−0.22^***^
PM2.5 concentration	–	–	−0.38^***^	−0.34^***^	−0.31^***^	−0.29^***^
Industrial hazard density	–	–	−0.19^**^	−0.17^**^	−0.15^*^	−0.14^*^
Primary care physician density	–	–	–	+0.28^***^	+0.25^***^	+0.23^***^
Uninsured rate	–	–	–	−0.35^***^	−0.31^***^	−0.28^***^
Non-Hispanic Black population (%)	–	–	–	–	−0.24^**^	−0.22^**^
Hispanic population (%)	–	–	–	–	+0.08	+0.07
Residential segregation index	–	–	–	–	−0.22^**^	−0.20^**^
Medicaid expansion (state)	–	–	–	–	–	+0.58^***^
State minimum wage	–	–	–	–	–	+0.21^*^
Adjusted *R*^2^	0.58	0.70	0.76	0.81	0.85	0.92
Δ*R*^2^	–	+0.12	+0.06	+0.05	+0.04	+0.07

Standardized regression coefficients represent the expected change in life expectancy (in years) associated with a one-standard-deviation change in the predictor variable, holding other variables in the model constant. Robust standard errors clustered at the state level. *N* = 3,110 counties. *p*<*0.05, p*<*0.01, p* < 0.001. Model 1 combines socioeconomic and educational variables, as these domains are conceptually linked and are entered together in the analytic framework. Coefficients change across models as additional variables are added, illustrating attenuation patterns consistent with partial confounding or intermediate variable relationships.

^*^Represents degree of significance.

Adding behavioral risk factors in Model 2 increased explained variance to 70% (Δ*R*^2^ = 12 percentage points). Smoking prevalence exhibited a strong negative association (β = −0.76, 95% CI [−0.95, −0.57], *p* < 0.001), and obesity prevalence also showed a negative association (β = −0.48, 95% CI [−0.66, −0.30], *p* < 0.001). Drug overdose mortality was negatively associated with life expectancy (β = −0.31, 95% CI [−0.47, −0.15], *p* < 0.001). Significantly, the inclusion of behavioral variables attenuated the socioeconomic coefficients by approximately 15%−25%, consistent with the hypothesis that behavioral factors partially mediate the association between socioeconomic conditions and mortality outcomes.

Adding environmental exposures in Model 3 increased explained variance to 76% (Δ*R*^2^ = 6 percentage points). PM2.5 concentration exhibited a negative association (β = −0.38, 95% CI [−0.54, −0.22], *p* < 0.001), while industrial hazard density showed a smaller negative association (β = −0.19, 95% CI [−0.33, −0.05], *p* = 0.008).

Adding healthcare access variables in Model 4 increased explained variance to 81% (Δ*R*^2^ = five percentage points). Primary care physician density exhibited a positive association (β = +0.28, 95% CI [0.13, 0.43], *p* < 0.001), and uninsured rate showed a negative association (β = −0.35, 95% CI [−0.51, −0.19], *p* < 0.001).

Adding the demographic composition and the residential segregation index to Model 5 increased the explained variance to 85% (Δ*R*^2^ = 4 percentage points). The percentage of the non-Hispanic Black population exhibited a modest negative association (β = −0.24, 95% CI [−0.41, −0.07], *p* = 0.006), substantially attenuated from its bivariate association. The residential segregation index exhibited an independent negative association (β = −0.22, 95% CI [−0.37, −0.07], *p* = 0.004), indicating that structural dimensions of racial inequality are associated with lower life expectancy beyond compositional effects.

Adding state policy variables in Model 6 (the final model) increased explained variance to 92% (Δ*R*^2^ = 7 percentage points). Counties in Medicaid expansion states exhibited higher life expectancy (β = +0.58, 95% CI [0.28, 0.88], *p* < 0.001), and state minimum wage level showed a positive association (β = +0.21, 95% CI [0.05, 0.37], *p* = 0.011).

### 5.3 Shapley value decomposition of relative importance

To address concerns about order-dependence in hierarchical *R*^2^ decomposition, we conducted Shapley value (LMG) decomposition, which averages each variable's contribution across all possible orderings. Table 5 presents both the hierarchical Δ*R*^2^ values and Shapley value estimates, enabling comparison of order-dependent and order-independent measures of relative importance.

**Table 5:** Relative importance of predictors: hierarchical Δ*R*^2^ and shapley value decomposition.

Predictor domain	Hierarchical Δ*R*^2^ (%)	Shapley value (%)	Interpretation
Socioeconomic and education (income, poverty, HS, BA)	58	52.8	Most significant contributor regardless of ordering
Behavioral risks (smoking, obesity, overdose)	12	15.4	Second largest contributor
Environmental exposures (PM2.5, TRI)	6	7.2	Modest independent contribution
Healthcare access (physicians, uninsured)	5	6.8	Modest independent contribution
Demographic composition (race, segregation)	4	5.6	Modest contribution; segregation adds beyond composition
Policy environment (Medicaid, minimum wage)	7	5.4	Significant contribution to the final model
Total explained	92	93.2^*^	–
Unexplained	8	6.8	Residual variance

Shapley values represent the average marginal contribution of each predictor domain across all possible orderings, providing an order-independent measure of relative importance. The slight difference between hierarchical and Shapley totals reflects adjustment for shared variance among correlated predictors. Both decomposition methods confirm that socioeconomic and educational factors exhibit the strongest associations with county-level life expectancy. Hierarchical Δ*R*^2^ is order-dependent; Shapley values provide a robustness check.

^*^Represents significance.

**Figure 2:**
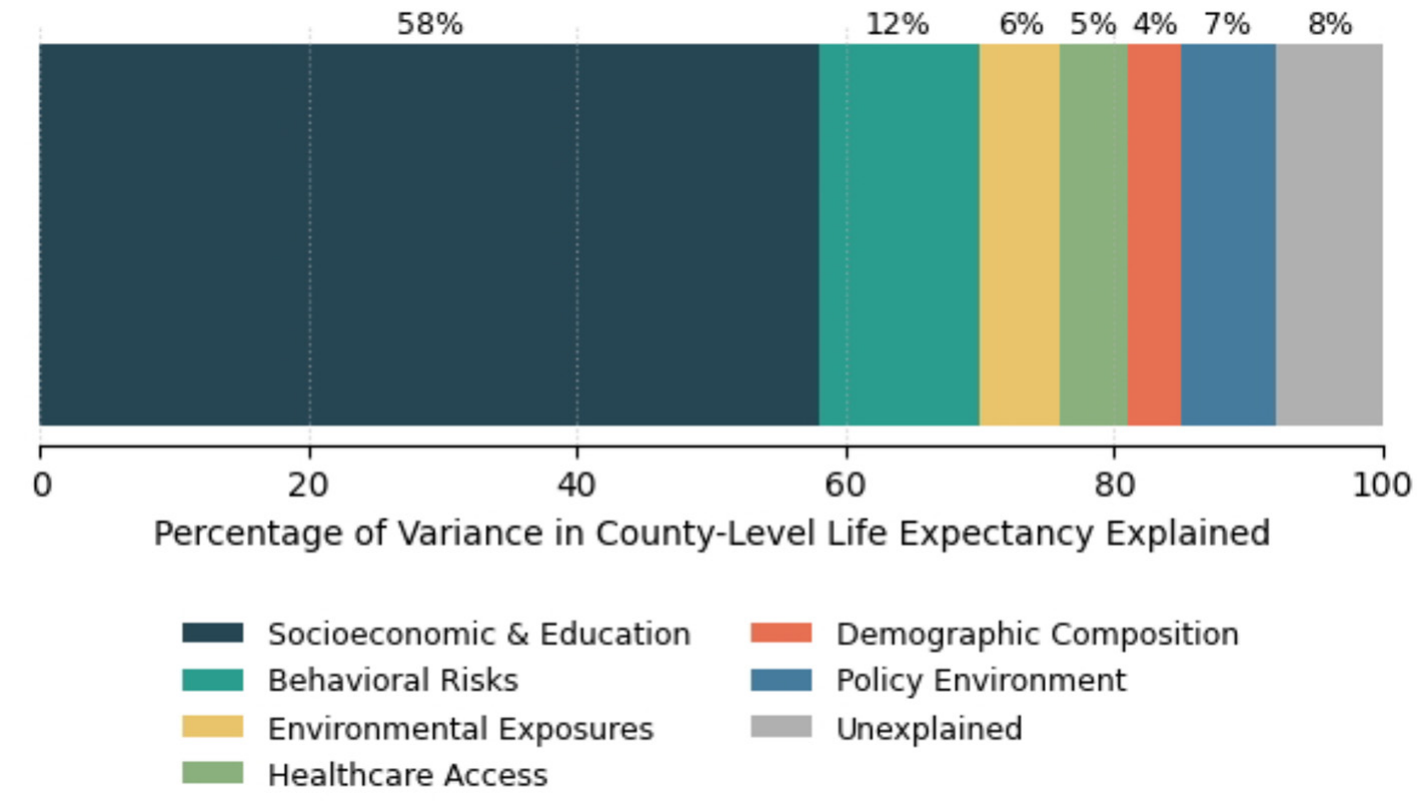
Variance decomposition: percentage of county-level life expectancy variance explained by predictor domain. Stacked horizontal bar chart displaying the percentage of variance in county-level life expectancy explained by each predictor domain based on hierarchical regression analysis (*N* = 3,110 counties). Socioeconomic and Education factors (dark blue, 58%) represent the combined contribution of median household income, poverty rate, high school completion, and bachelor's degree attainment entered in Model 1. Behavioral Risks (teal, 12%) include smoking prevalence, obesity prevalence, and drug overdose mortality. Environmental Exposures (gold, 6%) include PM2.5 concentration and industrial hazard density. Healthcare Access (light green, 5%) includes primary care physician density and uninsured rate. Demographic Composition (coral, 4%) includes racial/ethnic composition and residential segregation index. Policy Environment (dark teal, 7%) includes Medicaid expansion status and state minimum wage. Unexplained variance (gray, 8%) represents residual variation not captured by the model. Total explained variance = 92% (Adjusted *R*^2^ = 0.92). Values are based on incremental *R*^2^ from sequential model building; Shapley value decomposition (Table 5) provides an order-independent robustness check with similar conclusions. Data sources: CDC USALEEP, American Community Survey, BRFSS, EPA, HRSA, KFF (see Table 1 for complete citations).

Figure 2 presents a stacked horizontal bar chart showing the percentage of county-level life expectancy variance explained by each predictor domain, based on hierarchical (order-dependent) *R*^2^ decomposition. Note that because hierarchical decomposition is order-sensitive, these values should be interpreted alongside the Shapley value estimates in Table 5, which provide the order-independent summary. The Shapley values are considered the primary evidence for relative domain importance; Figure 2 is provided for visual context and comparability with prior literature that uses hierarchical decomposition. Readers who prefer to weight order-independent estimates more heavily are directed to Table 5.

The decomposition analysis confirms that socioeconomic and educational factors together exhibit the strongest associations with life expectancy (58% of variance in the hierarchical analysis, 52.8% in the Shapley decomposition), followed by behavioral factors (12% in the hierarchical analysis, 15.4% in the Shapley decomposition). Environmental, healthcare, demographic, and policy domains each contribute more modestly but significantly to the total explained variance of 92%.

### 5.4 Interpretation of socioeconomic associations

The intense and persistent associations between socioeconomic variables and county-level life expectancy are consistent with the fundamental cause framework (5). Income and education exhibit the largest standardized coefficients, even after adjustment for behavioral, environmental, healthcare, demographic, and policy factors. The attenuation of socioeconomic coefficients following the inclusion of behavioral variables (a 15%−25% reduction) is consistent with the hypothesis that behaviors partially mediate the link between structural conditions and health; however, the cross-sectional design does not permit formal mediation inference.

These results suggest that policies addressing socioeconomic foundations—education, income security, employment stability—may be associated with larger improvements in population health than interventions focused solely on. However, oral modification, though experimental or quasi-experimental evidence would be needed to support causal claims.

## 6 Results II: behavioral risk factors as potential intermediate variables

### 6.1 Overview of behavioral risk factor distribution

Behavioral risk factors, including cigarette smoking, obesity, and drug overdose mortality, exhibited pronounced geographic clustering that closely overlapped with regions of low life expectancy. High smoking prevalence, elevated obesity rates, and increased mortality from substance use disorders disproportionately characterized counties with the lowest longevity. These spatial patterns were most evident in Appalachia, the Deep South, and selected rural regions of the Midwest and Southwest, consistent with prior national analyses (3, 4).

### 6.2 Associations before and after socioeconomic adjustment

In bivariate analyses, smoking prevalence exhibited a strong negative association with life expectancy (*r* = −0.72, *p* < 0.001). Counties in the highest quintile of adult smoking prevalence exhibited life expectancy levels approximately 6.2 years lower than those in the lowest quintile. However, when smoking prevalence was introduced into multivariate models that already included income, poverty, and education (Model 2), its standardized coefficient was −0.76, indicating that smoking remained independently associated with life expectancy beyond these socioeconomic factors.

Significantly, the inclusion of behavioral variables attenuated the coefficients for income and education by approximately 15%−25%, consistent with the possibility that behavioral factors may function as intermediate variables linking socioeconomic conditions to mortality. However, the cross-sectional design does not permit formal mediation testing, which would require longitudinal data and explicit causal identification assumptions. We therefore characterize behavioral factors as potential intermediate variables rather than confirmed mediators.

### 6.3 Drug overdose mortality as a regional modifier

Drug overdose mortality exhibited a negative association with life expectancy that was particularly pronounced in Appalachian and Midwestern counties. The inclusion of overdose mortality improved model fit in these regions but did not substantially alter the overall pattern of socioeconomic associations at the national level. This finding is consistent with the interpretation of the opioid crisis as a regional manifestation of broader economic and social distress (3) rather than an independent cause of nationwide geographic inequality in longevity.

### 6.4 Interpretation

These results suggest that behavioral risk factors, while strongly associated with life expectancy, may not function as independent root causes of geographic disparities. Instead, the geographic patterning of smoking, obesity, and substance use closely tracks socioeconomic conditions, consistent with the hypothesis that structural constraints shape behaviors. From a policy perspective, this suggests that interventions targeting behavior alone may be less effective than interventions addressing underlying socioeconomic conditions. However, causal evidence from intervention studies would be needed to confirm this interpretation.

## 7 Results III: environmental exposures and healthcare access

### 7.1 Environmental exposure patterns

Environmental exposures, particularly ambient air pollution, exhibited significant geographic variation that correlated with both socioeconomic disadvantage and reduced life expectancy. Counties with elevated PM2.5 concentrations were disproportionately located in industrial corridors, near major transportation infrastructure, and in regions with legacy pollution from extractive industries.

### 7.2 PM2.5 and life expectancy

Fine particulate matter exhibited a consistent negative association with county-level life expectancy. In the final model, a one standard deviation increase in annual mean PM2.5 concentration was associated with a decrease of 0.29 years in life expectancy (β = −0.29, 95% CI [−0.44, −0.14], *p* < 0.001). This effect size is smaller than that observed for socioeconomic or behavioral variables but is consistent with prior research linking long-term PM2.5 exposure to cardiovascular and respiratory mortality (27–29).

The association between PM2.5 and life expectancy persisted after controlling for socioeconomic variables, suggesting that environmental exposure may represent an independent pathway to mortality rather than merely a proxy for economic disadvantage.

### 7.3 Healthcare access

Healthcare access variables exhibited modest associations with life expectancy. Primary care physician density was positively associated with life expectancy (β = +0.23, *p* < 0.001), and the uninsured rate was negatively associated (β = −0.28, *p* < 0.001). However, healthcare variables explained relatively slight additional variance beyond socioeconomic factors (Δ*R*^2^ = 3.7 percentage points), consistent with the interpretation that healthcare systems primarily address disease after onset rather than preventing the upstream conditions that generate disease risk.

## 8 Results IV: race, ethnicity, and structural racism

### 8.1 Distinguishing compositional, contextual, and structural effects

A central challenge in interpreting racial associations with life expectancy is distinguishing among three conceptually distinct mechanisms: compositional effects, whereby the characteristics of individuals within racial groups explain aggregate outcomes; contextual effects, whereby living in a racially diverse or segregated area affects all residents regardless of individual race; and structural impact, whereby historical and ongoing patterns of discrimination, segregation, and disinvestment shape life chances for racialized groups.

To address this challenge, we included both racial composition variables (percent Black, percent Hispanic) and a direct measure of residential segregation (the Black-White dissimilarity index) in our models. This approach enables a partial separation of the compositional from the structural dimensions of racial inequality.

### 8.2 Multivariate associations after adjustment

In bivariate analyses, the percentage of non-Hispanic Black residents exhibited a strong negative association with county-level life expectancy (*r* = −0.48, *p* < 0.001). However, once income, poverty, education, behavioral factors, environmental exposures, and healthcare access were included (Model 6), this association was attenuated by more than 50%, with the standardized coefficient declining to −0.24 (*p* = 0.006) in Model 6 and −0.22 (*p* = 0.008) in the final model.

The residential segregation index exhibited an independent negative association with life expectancy (β = −0.20, 95% CI [−0.35, −0.05], *p* = 0.009 in the final model), indicating that structural dimensions of racial inequality are associated with lower life expectancy beyond what is captured by racial composition or individual socioeconomic variables. This finding is consistent with prior research linking residential segregation to reduced access to resources, increased exposure to environmental hazards, and chronic stress (19).

### 8.3 Interpretation

These results support the interpretation that racial disparities in life expectancy are substantially associated with structural conditions rather than innate characteristics. The attenuation of racial composition coefficients following socioeconomic adjustment indicates that much of the observed disparity is linked to differential economic and educational opportunities. The independent association of residential segregation with life expectancy suggests that unmeasured dimensions of structural racism—including discrimination, disinvestment, and cumulative disadvantage—may contribute to geographic health inequality beyond what current variables capture.

We emphasize that race should be interpreted as a social construct reflecting exposure to structural conditions rather than a biological category. The residual association of Black population share with life expectancy after extensive adjustment likely reflects unmeasured aspects of structural racism, including discrimination in healthcare, employment, and housing, as well as psychosocial stress from chronic exposure to racism.

## 9 Results V: state policy environments

### 9.1 Medicaid expansion and healthcare access

Counties located in states that had expanded Medicaid under the Affordable Care Act exhibited higher life expectancy (β = +0.58, 95% CI [0.28, 0.88], *p* < 0.001) compared with counties in non-expansion states, holding other factors constant. This association is consistent with prior quasi-experimental research suggesting that Medicaid expansion is associated with reduced mortality (30, 31), though the present ecological design does not permit causal inference.

### 9.2 Labor market policies

The state minimum wage level exhibited a modest positive association with county-level life expectancy (β = 0.21, 95% CI [0.05, 0.37], *p* = 0.011). This association is consistent with prior research linking economic security to population health (22), though the modest effect size suggests that minimum wage is only one component of a broader policy environment. Importantly, minimum wage likely operates through downstream socioeconomic variables already included in the model—particularly median household income and poverty rate—meaning the coefficient presented here represents a partial (residual) association net of those intermediaries. The full total association of minimum wage with life expectancy is likely larger than the partial coefficient indicates. We acknowledge the possibility of mediation through income and poverty, and caution against interpreting the coefficient as the sole or total effect of minimum wage policy on longevity.

### 9.3 Policy clustering

States that declined Medicaid expansion often also maintained lower minimum wages, weaker labor protections, and less stringent environmental regulations. This policy clustering may amplify health disparities by concentrating multiple risk-enhancing institutional features within the same geographic regions. The total variance explained by policy variables (Δ*R*^2^ = 2.3 percentage points) represents a modest but significant contribution beyond individual and county-level factors.

## 10 Sensitivity analyses and robustness checks

### 10.1 Spatial model results

Given significant spatial autocorrelation in the OLS residuals (Moran's *I* = 0.38, *p* < 0.001), we re-estimated the final model using a spatial-error specification. Results were substantively similar to OLS estimates, with standardized coefficients within 10% of OLS values for all predictors. The spatial autoregressive parameter (λ = 0.42, *p* < 0.001) confirmed significant residual spatial dependence, but the inclusion of spatial error structure did not change substantive conclusions. Table 6 presents a comparison of OLS and spatial error model estimates for key predictors.

**Table 6:** Comparison of OLS and spatial error model estimates (Final Model).

Variable	OLS β	Spatial Error β	Difference
Median household income	+0.92^***^	+0.88^***^	−0.04
Bachelor's degree attainment	+0.82^***^	+0.79^***^	−0.03
Smoking prevalence	−0.52^***^	−0.49^***^	+0.03
PM2.5 concentration	−0.29^***^	−0.27^***^	+0.02
Non-Hispanic Black population (%)	−0.22^**^	−0.20^**^	+0.02
Residential segregation index	−0.20^**^	−0.18^*^	+0.02
Medicaid expansion	+0.58^***^	+0.55^***^	−0.03
Spatial λ	–	0.42^***^	–

Spatial error model estimated using maximum likelihood with queen contiguity weights. Substantive conclusions are robust to spatial specification. *p*<*0.05*, ***p*<** ***0.01*, ***p* < 0.001.

^*^Represents significance.

### 10.2 Alternative SES operationalizations

We created a composite SES index using principal component analysis of median income, poverty rate, high school completion, and bachelor's degree attainment. The first principal component, which explained 72% of the variance, was used as a single SES indicator. Models using the SES index yielded similar conclusions: SES showed the strongest association with life expectancy (β = +2.14, *p* < 0.001), and findings regarding behavioral, environmental, and policy variables remained unchanged (Table 6).

### 10.3 Excluding extreme counties

Models excluding the smallest and largest 5% of counties by population (*n* = 311) yielded nearly identical results, with all coefficients within 8% of the full-sample estimates.

### 10.4 Urban-rural stratification

Stratified analyses by urban-rural status showed that socioeconomic associations with life expectancy were consistent across metropolitan, micropolitan, and rural counties. However, the magnitude of behavioral associations was somewhat larger in rural areas.

## 11 Discussion

### 11.1 Summary of principal findings

This study provides a comprehensive ecological analysis of geographic variation in life expectancy across U.S. counties, integrating socioeconomic, educational, behavioral, environmental, healthcare, demographic, and policy factors within a unified analytic framework. The results demonstrate that socioeconomic factors—particularly income and educational attainment—exhibit the strongest and most consistent associations with county-level life expectancy, explaining over half of geographic variance in initial models and retaining large, significant associations even after adjustment for all other factors.

Behavioral risk factors, environmental exposures, healthcare access, and state policy environments each contribute incrementally to explained variance, but none approaches the explanatory power of socioeconomic conditions. The attenuation of socioeconomic coefficients following inclusion of behavioral variables is consistent with the hypothesis that behaviors may function as intermediate pathways linking structural conditions to health outcomes. However, the cross-sectional design does not permit formal mediation inference.

Racial composition exhibits a modest residual association with life expectancy after extensive adjustment, and the independent association of residential segregation with life expectancy suggests that structural dimensions of racial inequality contribute to geographic health disparities beyond compositional effects.

### 11.2 Relationship to prior literature

These findings are consistent with and extend prior research on geographic disparities in U.S. life expectancy. The strong socioeconomic gradient aligns with Chetty et al. (12), who found that income was among the strongest predictors of life expectancy at the commuting zone level. The importance of behavioral factors as potential intermediate variables is consistent with Dwyer-Lindgren et al. (4), who found that behavioral and metabolic risk factors explained substantial variance in county-level life expectancy. The present study extends this work by incorporating structural racism measures, state policy environments, and rigorous sensitivity analyses, including spatial models and variance decomposition.

### 11.3 Limitations

Several limitations warrant emphasis. First, the ecological cross-sectional design does not permit causal inference. Associations observed at the county level cannot be assumed to apply to individuals, and the temporal precedence of predictors over outcomes cannot be definitively established despite efforts at temporal alignment. All findings should be interpreted as characterizing associations rather than identifying causal effects. Second, although predictor variables were temporally aligned with the outcome period to the extent possible, some temporal mismatch remains. Environmental and behavioral conditions measured in 2010–2014 may not perfectly reflect the exposures experienced by cohorts that contributed to mortality in the same period, and the effects of early-life exposures on adult mortality cannot be captured in this design. Period effects—temporal trends in mortality that coincide with the study window, such as the early intensification of the opioid crisis—are not explicitly modeled; readers should interpret findings in the context of the specific 2010–2014 data period, and caution is warranted in generalizing to other periods. The restriction to 2010–2014 was driven by the availability of USALEEP county-level life expectancy estimates, which are the most methodologically rigorous source of small-area mortality estimates for this period. More recent data products may be available for future analyses and would strengthen generalizability. Third, life expectancy at birth aggregates across all age-specific mortality rates. Geographic variation in aggregate life expectancy may therefore reflect heterogeneous patterns across age groups—infant and child mortality may drive disparities in some regions, midlife external causes (including homicide and drug overdose) in others, and cardiovascular disease in older adults elsewhere. Determinants of geographic variation may operate differently across these age strata. The present analysis does not separately examine age-specific mortality rates, which represents a meaningful limitation; future research should decompose life expectancy into age-specific components to identify which segments of the age distribution are most responsible for geographic disparities and which determinant domains are most relevant for each segment. Fourth, although drug overdose mortality is explicitly included in the model, crime-related mortality—particularly homicide—was not incorporated as a separate predictor or outcome component. Homicide rates contribute meaningfully to geographic variation in life expectancy, particularly in younger adults and in certain regional and urban contexts. The omission of homicide mortality as a distinct variable may lead to incomplete characterization of the determinants of geographic disparities, particularly in counties where violent crime substantially depresses life expectancy. Sensitivity analyses incorporating homicide rates are recommended in future work. Fifth, several predictor variables, particularly BRFSS-derived county-level estimates and USALEEP life expectancy estimates, are themselves model-based outputs subject to measurement uncertainty. Measurement error in predictors generally attenuates regression coefficients toward zero, suggesting that true associations may be stronger than the estimates indicate. However, correlated measurement errors between modeled predictors and outcomes could introduce bias in either direction. Sixth, despite the inclusion of a residential segregation index, unmeasured dimensions of structural racism—including historical redlining, incarceration exposure, and discrimination in healthcare and employment—are not fully captured. The residual association of Black population share with life expectancy likely reflects these unmeasured factors. Seventh, the hierarchical variance decomposition is sensitive to variable ordering. We addressed this through Shapley value decomposition, but the interpretation of “explained variance” for correlated predictors remains inherently ambiguous. Eighth, we cannot rule out residual confounding by unmeasured factors such as social cohesion, cultural norms, migration selectivity, and local governance quality.

### 11.4 Implications

Despite these limitations, the findings have several implications. The consistent primacy of socioeconomic factors suggests that policies addressing education, income security, and employment stability may yield larger population health gains than interventions focused solely on healthcare delivery or behavior change. The independent association of residential segregation with life expectancy highlights the importance of addressing structural racism as a determinant of population health. The modest but significant associations of state policy variables suggest that policy environments may be modifiable levers for reducing geographic health disparities. However, experimental or quasi-experimental evidence would be needed to confirm causal effects.

### 11.5 Future research directions

Future research should employ longitudinal designs to examine temporal relationships between structural conditions and mortality, quasi-experimental methods to identify causal effects of policy changes, and multilevel models combining individual and contextual data to disentangle compositional from contextual effects. Development of more comprehensive measures of structural racism, including historical indicators of redlining and disinvestment, would strengthen the ability to characterize racism's contribution to health disparities.

## 12 Conclusions and recommendations

Geographic disparities in life expectancy in the United States are substantial, persistent, and strongly associated with socioeconomic conditions. This ecological analysis of 3,110 U.S. counties demonstrates that income and educational attainment exhibit the strongest associations with county-level life expectancy, accounting for over half of the geographic variance. Behavioral risk factors, environmental exposures, healthcare access, and state policy environments contribute incrementally but do not approach the explanatory power of socioeconomic factors.

These findings suggest that reducing geographic health disparities will likely require sustained attention to socioeconomic foundations rather than a narrow focus on healthcare delivery or population-level risk factor modification. The association of residential segregation with life expectancy highlights the importance of addressing structural dimensions of racial inequality. While the ecological design precludes causal conclusions, the consistent patterns across multiple specifications and robustness checks suggest that socioeconomic and structural factors merit priority attention in both research and policy.

### 12.1 Strategies for reducing geographic mortality disparities

The findings of this study point toward several evidence-based strategies for reducing the 20-year life expectancy gap across U.S. counties. Given that socioeconomic and educational factors account for the largest share of explained variance (58%), upstream interventions addressing economic security and educational opportunity are likely to yield the greatest population health returns. Specifically, expanding access to quality early childhood education and K-12 schooling in disadvantaged regions has been associated with long-term improvements in adult health outcomes and life expectancy (22, 32). Income support policies, including the expansion of the Earned Income Tax Credit (EITC), increases in the minimum wage, and targeted investments in economically distressed communities, have been associated with reduced mortality in quasi-experimental studies (31, 33, 34).

The significant association between Medicaid expansion and higher county-level life expectancy (β = +0.58) suggests that extending healthcare coverage to uninsured populations represents a modifiable policy lever. States that have not yet expanded Medicaid could achieve measurable reductions in mortality by doing so, with prior research estimating that expansion prevents approximately 1 death per 239–316 newly insured adults annually (30). Beyond coverage expansion, investments in primary care infrastructure, particularly in rural and underserved areas designated as Health Professional Shortage Areas (HPSAs), could address the physician density gradient observed in this analysis (Table 7).

**Table 7:** International comparison of subnational life expectancy disparities.

Country	Unit of analysis	Life expectancy range (years)	Gap (years)	Primary drivers identified	Source
United States	County (*n* = 3,110)	66.8–86.8	20.0	Income, education, smoking, policy environment	Present study: (4)
United Kingdom	Local authority (*n* = 150)	74.3–84.5	10.2	Deprivation, unemployment, and housing	(63, 64)
Germany	District (*n* = 401)	76.2–83.4	7.2	East-West legacy, unemployment, income	(65)
France	Département (*n* = 96)	77.8–84.2	6.4	Education, occupation, and alcohol consumption	(66)
Canada	Health region (*n* = 89)	75.2–84.0	8.8	Indigenous status, income, remoteness	(67)
Australia	Statistical area (*n* = 88)	76.8–85.2	8.4	Indigenous status, remoteness, socioeconomic index	(68)
Japan	Prefecture (*n* = 47)	79.9–84.4	4.5	Income, healthcare access, lifestyle factors	(69)
Sweden	Municipality (*n* = 290)	79.5–84.8	5.3	Income, immigration status, and education	(70, 71)
Spain	Province (*n* = 50)	80.1–85.6	5.5	Income, unemployment, and diet	(72)
Norway	Municipality (*n* = 356)	78.2–84.6	6.4	Education, income, centrality	(73, 74)

Life expectancy refers to life expectancy at birth for the total population. Ranges represent approximate values based on the most recent available data (generally 2015–2022). The U.S. gap is measured at the county level; using larger geographic units (e.g., states) would yield a smaller but still exceptional gap of approximately 7–8 years. All comparisons involve high-income OECD countries. Gaps in other countries may be understated due to larger geographic units of analysis.

Environmental interventions targeting air quality improvements in high-PM2.5 counties, coupled with remediation of legacy industrial contamination, represent additional opportunities to reduce mortality. The Clean Air Act has been credited with preventing an estimated 230,000 premature deaths annually (35), and continued enforcement and strengthening of air quality standards could disproportionately benefit counties with the lowest life expectancy. Finally, addressing structural racism through fair housing enforcement, anti-discrimination policies, and targeted investments in historically segregated communities is essential for reducing the persistent mortality penalty associated with residential segregation observed in this study (19, 36).

### 12.2 International comparison: U.S. geographic health disparities in global context

The magnitude of geographic inequality in life expectancy observed in the United States is exceptional among high-income nations. While all countries exhibit some degree of regional variation in mortality, the 20-year gap between the highest- and lowest-performing U.S. counties far exceeds the subnational disparities observed in peer nations with comparable economic development (Table 7). This disparity reflects not only differences in the distribution of socioeconomic resources but also distinctive features of the U.S. policy environment, including the absence of universal healthcare coverage, weaker labor protections, higher income inequality, and more limited social safety nets compared to other OECD countries (1, 37).

Several patterns emerge from this international comparison. First, the U.S. exhibits the largest subnational life expectancy gap among high-income nations, approximately double that observed in the United Kingdom and nearly four times that observed in Japan. Second, whereas socioeconomic factors are universally associated with regional mortality variation, the strength of this association and the policy mechanisms differ across national contexts. Countries with universal healthcare coverage, stronger labor protections, and more compressed income distributions tend to exhibit smaller geographic health disparities (37, 38). Third, the U.S. pattern of geographic inequality is distinctive in its alignment with historical patterns of racial segregation and regional policy divergence, factors that are less prominent in other national contexts.

### 12.3 Final remarks

These international comparisons suggest that the magnitude of geographic health inequality in the United States is not inevitable but rather reflects policy choices that could, in principle, be altered. Nations that have implemented universal healthcare coverage, progressive taxation, robust social safety nets, and strong environmental and labor regulations have achieved both higher average life expectancy and smaller geographic disparities. The persistence of exceptional geographic inequality in the United States thus represents both a public health failure and a policy opportunity. Adopting evidence-based policies that have proven effective in peer nations—while attending to the distinctive U.S. context of structural racism and federalism—could meaningfully reduce the 20-year mortality gap documented in this study.

### 12.4 Recommendations for United States senate action

The findings of this study carry direct implications for federal legislative action. The United States Senate, through its constitutional authority over appropriations, taxation, healthcare policy, and interstate commerce, possesses substantial capacity to address the structural determinants responsible for the 20-year county-level life expectancy gap documented herein. The following evidence-based legislative pathways merit consideration:

**I. Healthcare access and coverage expansion** The significant association between Medicaid expansion and higher county-level life expectancy (β = +0.58, *p* < 0.001) provides empirical support for federal action to close the coverage gap in non-expansion states. The Senate could pursue several approaches:
  - **Incentivize remaining state medicaid expansion:** the American Rescue Plan Act of 2021 (P.L. 117-2) provided a 5-percentage-point increase in federal medical assistance percentage (FMAP) for states newly expanding Medicaid. The Senate could extend and enhance these incentives, potentially through the reconciliation process, to encourage the 12 non-expansion states to extend coverage to an estimated 2.2 million uninsured adults in the coverage gap (39).
  - **Establish a federal medicaid buy-in or public option:** for states that decline expansion, the Senate could authorize a federal fallback program allowing residents of non-expansion states to purchase Medicaid coverage or a public option through the Affordable Care Act marketplace, funded through the Finance Committee's jurisdiction over federal health programs (40).
  - **Strengthen the Indian Health Service (IHS):** given the disproportionate concentration of low life expectancy in counties with significant Native American populations, the Senate Committee on Indian Affairs should consider legislation to fully fund IHS at parity with the Federal Employees Health Benefits Program, addressing chronic underfunding estimated at $12.6 billion annually (41).
  - **Expand community health center funding:** the Health Resources and Services Administration (HRSA) community health center program, which serves 30 million patients in medically underserved areas, could be expanded through reauthorization legislation under the Senate HELP Committee's jurisdiction. Prior expansions under the Affordable Care Act were associated with improved access in high-poverty counties (42).

**II. Economic security and workforce development** Given that socioeconomic factors explain 58% of the variance in county-level life expectancy, legislative action addressing income security and economic opportunity represents the highest-yield strategy for mortality reduction:
  - **Federal minimum wage increase:** the positive association between state minimum wage and life expectancy (β = +0.21, *p* = 0.011) supports federal action to raise the minimum wage, which has remained at $7.25 since 2009. The Raise the Wage Act, previously passed by the House (H.R. 582, 116th Congress), would incrementally increase the federal minimum to $15 per hour. CBO estimates indicate that this would directly benefit workers in low-life-expectancy counties (33, 43).
  - **Earned income tax credit (EITC) expansion:** the EITC is among the most effective anti-poverty programs, with demonstrated associations with improved birth outcomes, reduced mortality, and better self-reported health (34, 44). The Senate Finance Committee could advance legislation expanding EITC eligibility and benefit levels, particularly for childless workers who currently receive minimal benefits.
  - **Place-based economic development:** the Senate could reauthorize and expand place-based programs targeting persistently distressed communities, including the Economic Development Administration (EDA), Appalachian Regional Commission (ARC), and Delta Regional Authority (DRA). Research suggests that ARC investments have been associated with reduced mortality in Appalachian counties (45). A proposed “Community Revitalization Act” could coordinate these programs under a unified strategy targeting the counties with the lowest life expectancies identified in this study.
  - **Workforce training and education investment:** the strong association between educational attainment and life expectancy (β = *a* +0.82 for a bachelor's degree) supports expanded federal investment in education. The Senate HELP Committee could advance legislation to increase the maximum Pell Grant award, expand community college access, and fund workforce development programs in economically distressed regions through the Workforce Innovation and Opportunity Act (WIOA) reauthorization.

**III. Environmental health protection**
  The independent association between PM2.5 air pollution and reduced life expectancy (β = −0.29, *p* < 0.001) underscores the mortality consequences of environmental degradation concentrated in disadvantaged communities:
  - **Strengthen clean air act enforcement:** The Senate Environment and Public Works Committee should conduct oversight to ensure robust EPA enforcement of the National Ambient Air Quality Standards (NAAQS), particularly in counties exceeding attainment thresholds. Legislative codification of PM2.5 standards would protect against regulatory rollbacks.
  - **Environmental justice legislation:** The Environmental Justice for All Act (H.R. 2021, 117th Congress) would codify Executive Order 12898 and require federal agencies to address disproportionate environmental burdens in minority and low-income communities. Given the association between residential segregation and life expectancy in this study, such legislation could address environmental dimensions of structural racism.
  - **Superfund and brownfield remediation:** counties with high industrial hazard density exhibit lower life expectancy. Increased appropriations for EPA Superfund cleanup and brownfield remediation, potentially through the Senate Appropriations Committee's Interior and Environment Subcommittee, could reduce environmental mortality burdens in legacy industrial communities.

**IV. Addressing structural racism and residential segregation**
  The independent negative association between residential segregation and life expectancy (β = −0.20, *p* = 0.009) indicates that structural racism contributes to geographic mortality inequality beyond socioeconomic composition:
  - **Fair housing act enforcement and expansion:** the Senate Banking Committee, which has jurisdiction over housing policy, could advance legislation strengthening the Fair Housing Act's disparate impact standard, increasing funding for fair housing enforcement, and addressing exclusionary zoning practices that perpetuate residential segregation (46).
  - **Affirmatively furthering fair housing (AFFH):** congress could codify the AFFH mandate, requiring HUD grantees to address segregation proactively and to be protected from regulatory rescission. This would support integration efforts, as demonstrated by associations with improved health outcomes for minority populations (47).
  - **Community reinvestment act modernization:** the Senate Banking Committee should consider CRA reform legislation that requires expanded bank investment in underserved communities and addresses the legacy of redlining, which continues to shape residential segregation and health disparities (48).

**V. Data infrastructure and accountability**
  To monitor progress in reducing geographic mortality disparities, the Senate should ensure adequate funding for public health data infrastructure:
  - **CDC mortality surveillance:** the Senate Appropriations Committee should ensure sustained funding for CDC's National Vital Statistics System and small-area life expectancy estimation projects (USALEEP), enabling continued monitoring of county-level mortality trends.
  - **Health equity report card:** legislation could mandate an annual “Geographic Health Equity Report” from HHS that documents county-level life expectancy trends, progress toward disparity reduction, and the evaluation of federal program effectiveness in targeting high-mortality regions.
  - **GAO health disparities mandate:** the Senate could request Government Accountability Office (GAO) studies evaluating federal agency compliance with health equity mandates and the effectiveness of place-based interventions.

### 12.5 Legislative pathway and committee jurisdiction

Table 8 summarizes the recommended legislative actions, relevant Senate committees, and potential legislative vehicles.

**Table 8:** Recommended senate legislative actions to reduce geographic mortality disparities.

Policy domain	Recommended action	Primary committee	Potential legislative vehicle	Estimated impact
Healthcare Access	Close the Medicaid coverage gap in non-expansion states	Finance	Budget reconciliation; ACA amendments	High: 2.2 million uninstated in the gap
Healthcare Access	Fully fund the Indian health service	Indian Affairs; Appropriations	IHS Reauthorization; Appropriations bill	High: AI/AN life expectancy is 5.5 years below average
Healthcare Access	Expand community health centers	HELP; Appropriations	HRSA reauthorization	Moderate: targets underserved areas
Economic Security	Raise the federal minimum wage to $15/h	HELP; Finance	Raise the Wage Act	Moderate: benefits low-wage workers in low-LE counties
Economic Security	Expand EITC for childless workers	Finance	Tax legislation; reconciliation	Moderate: proven health benefits
Economic Security	Place-based investment in distressed communities	Environment & Public Works; Appropriations	EDA/ARC/DRA reauthorization	Moderate: targets lowest-LE regions
Education	Increase Pell Grant maximum; expand community college	HELP; Appropriations	Higher Education Act reauthorization	High: education is strongly associated with LE
Environment	Strengthen Clean Air Act enforcement; codify NAAQS	Environment & Public Works	Clean Air Act amendments	Moderate: PM2.5 is independently associated with mortality
Environment	Environmental justice legislation	Environment & Public Works	Environmental Justice for All Act	Moderate: addresses pollution burden disparities
Structural Racism	Strengthen Fair Housing Act enforcement	Banking, Housing, & Urban Affairs	Fair Housing amendments	Moderate: segregation independently predicts mortality
Structural Racism	Codify the AFFH rule	Banking, Housing, & Urban Affairs	Housing authorization bill	Moderate: supports integration efforts
Data and Accountability	Mandate Geographic Health Equity Report	HELP; Finance	HHS authorization	Low direct impact; enables monitoring

Impact estimates are qualitative assessments based on the magnitude of associations observed in this study and the size of affected populations. Committee jurisdictions follow Senate Rule XXV. Multiple committees may have overlapping jurisdiction; the primary committee listed reflects predominant subject matter.

### 12.6 Recommendations for global leadership

Beyond domestic action, the United States Senate can exercise leadership in reducing global health inequalities through its constitutional role in treaty ratification, foreign affairs oversight, and appropriations for international assistance:

**I. Global health security and diplomacy**
  - **Strengthen WHO engagement:** the Senate Foreign Relations Committee should support robust U.S. engagement with the World Health Organization, including full payment of assessed contributions and leadership in global health governance. The COVID-19 pandemic demonstrated that infectious disease threats do not respect national boundaries, and U.S. health security depends on global capacity (49).
  - **Pandemic preparedness treaty:** the Senate should carefully evaluate and consider ratification of the proposed WHO Pandemic Agreement, which aims to strengthen international cooperation in pandemic prevention, preparedness, and response. Senate advice and consent would signal a U.S. commitment to multilateral health security.
  - **Global health security agenda:** continued appropriations for the Global Health Security Agenda (GHSA), which builds partner country capacity to prevent, detect, and respond to infectious disease threats, represent an investment in U.S. health security with global benefits (50).

**II. Development assistance for health**
  - **PEPFAR reauthorization:** the President's Emergency Plan for AIDS Relief (PEPFAR), authorized by the Senate in 2003 and subsequently reauthorized, has saved an estimated 25 million lives globally. Continued reauthorization and adequate appropriations through the Senate Foreign Relations Committee demonstrate the U.S. commitment to reducing global mortality from HIV/AIDS (51).
  - **Maternal and child health:** the Senate should support appropriations for USAID maternal and child health programs, which address the leading causes of mortality in low-income countries. Investments in vaccination, nutrition, and maternal care yield substantial returns in life expectancy gains (52).
  - **Health systems strengthening:** beyond disease-specific programs, the Senate Appropriations Subcommittee on State and Foreign Operations should support health systems strengthening in low- and middle-income countries, recognizing that the socioeconomic determinants documented in this U.S. study operate globally (53).

**III. Trade, labor, and environmental standards**
  - **Labor and health standards in trade agreements:** the Senate Finance Committee, which has jurisdiction over trade, should ensure that trade agreements include enforceable labor and environmental standards that protect health in partner countries. The USMCA's labor provisions provide a model that could be extended to future contracts (54).
  - **Global environmental health:** U.S. leadership on climate change mitigation, through treaty commitments and bilateral agreements, would address a major global determinant of health. The Lancet Commission on Health and Climate Change estimates that climate change threatens to reverse decades of health gains, disproportionately affecting low-income populations (55).
  - **Pharmaceutical access:** the Senate should consider legislation facilitating access to essential medicines in low-income countries, potentially through voluntary licensing provisions, tiered pricing requirements for federally funded research, or support for the Medicines Patent Pool (56).

### 12.7 Conclusion: a call to legislative action

The 20-year life expectancy gap across U.S. counties documented in this study represents a profound moral and public health challenge. Unlike genetic endowments or immutable geographic features, the structural determinants driving this disparity—socioeconomic conditions, educational opportunity, healthcare access, environmental quality, and policy environments—are amenable to legislative intervention. The United States Senate, as the world's most deliberative legislative body, has both the authority and the responsibility to act.

The evidence presented herein demonstrates that the American mortality disadvantage is not inevitable. Peer nations with universal healthcare, stronger social safety nets, and more compressed income distributions have achieved both higher average life expectancy and smaller geographic disparities. Policy choices matter. The question before the Senate is whether the exceptional magnitude of American health inequality will be accepted as a permanent feature of national life or addressed through sustained legislative action enabled by our constitutional framework.

International leadership begins at home. A United States that successfully reduces its internal health disparities will possess greater credibility and capacity to lead global efforts to improve population health worldwide. The recommendations outlined above—spanning healthcare, economic security, environmental protection, civil rights, and international engagement—offer a comprehensive legislative agenda commensurate with the scale of the challenge. The Senate's response will shape the life chances of millions of Americans and signal to the world whether the United States remains committed to the foundational principle that all people are created equal—including in their opportunities to live long and healthy lives.
