# Supplementary material for: The geography of American longevity: structural determinants of a 20-year life expectancy divide and a federal policy blueprint
Source: Front Public Health. 2026 Apr 28;14:1784769. doi: 10.3389/fpubh.2026.1784769 (PMC13160854; doi:10.3389/fpubh.2026.1784769)
Supplement: Supplementary file 1 [file Supplementary_file_1.docx]

# Appendix A: Supplementary Model Specifications

Table A1 presents the complete model specifications for each step in the hierarchical regression analysis, including the variables included and the functional form of each model.

Table A1. Full Model Specifications by Hierarchical Step

| Model | Variables Included | Functional Form |
| --- | --- | --- |
| 1 | Median income (log-transformed), Poverty rate, HS completion, BA attainment | LE = β₀ + β₁ln(Income) + β₂Poverty + β₃HS + β₄BA + ε |
| 2 | Model 1 + Smoking, Obesity, Overdose mortality | LE = Model 1 + β₅Smoking + β₆Obesity + β₇Overdose + ε |
| 3 | Model 2 + PM2.5, TRI density | LE = Model 2 + β₈PM2.5 + β₉TRI + ε |
| 4 | Model 3 + Physician density, Uninsured rate | LE = Model 3 + β₁₀Physicians + β₁₁Uninsured + ε |
| 5 | Model 4 + %Black, %Hispanic, %Age65+, Segregation index | LE = Model 4 + β₁₂Black + β₁₃Hispanic + β₁₄Age65 + β₁₅Segregation + ε |
| 6 | Model 5 + Medicaid expansion, Minimum wage, Union density | LE = Model 5 + β₁₆Medicaid + β₁₇MinWage + β₁₈Union + ε |

*Note:* Model 1 combines socioeconomic and education variables as the baseline model, consistent with the conceptual framework treating these as jointly determined upstream determinants. All continuous predictors were standardized (mean = 0, SD = 1) before estimation. Robust standard errors clustered at the state level (51 clusters, including DC). LE = Life expectancy at birth.

# Appendix B: Data Sources, Analytical Resources, and Platforms for Further Study

This appendix provides a comprehensive guide to publicly available data sources, analytical platforms, and research resources relevant to geographic disparities in life expectancy and population health. These resources enable independent verification of findings, state- and county-level analysis, and ongoing monitoring of mortality trends.

#### B.1 Life Expectancy and Mortality Data

| **Resource** | **Description** | **Geographic Level** | **URL** |
| --- | --- | --- | --- |
| CDC USALEEP | U.S. Small-Area Life Expectancy Estimates Project; model-based county-level life expectancy estimates | Census tract, County | <https://www.cdc.gov/nchs/nvss/usaleep/usaleep.html> |
| CDC WONDER | Wide-ranging Online Data for Epidemiologic Research; mortality data by cause, age, race, geography | County, State, National | <https://wonder.cdc.gov/> |
| IHME Global Burden of Disease | County-level life expectancy and mortality estimates with historical trends (1980–present) | County, State, National, Global | <https://www.healthdata.org/research-analysis/gbd> |
| NVSS Mortality Data | National Vital Statistics System; underlying mortality microdata | County, State, National | <https://www.cdc.gov/nchs/nvss/deaths.htm> |
| Social Security Area Population Projections | Life tables and mortality projections | National | <https://www.ssa.gov/oact/NOTES/as120/LifeTables_Tbl_7.html> |
| Human Mortality Database | International mortality and life expectancy data for comparative analysis | National (40+ countries) | <https://www.mortality.org/> |

#### B.2 Socioeconomic and Demographic Data

| **Resource** | **Description** | **Geographic Level** | **URL** |
| --- | --- | --- | --- |
| American Community Survey (ACS) | Income, poverty, education, insurance, housing, employment data | Census tract, County, State | <https://www.census.gov/programs-surveys/acs> |
| Census Bureau Data Explorer | Interactive access to Census and ACS data | All levels | <https://data.census.gov/> |
| Bureau of Economic Analysis (BEA) | Regional economic accounts, GDP, personal income | County, State, MSA | <https://www.bea.gov/data/economic-accounts/regional> |
| Bureau of Labor Statistics (BLS) | Employment, unemployment, wages, labor force data | County, State, MSA | <https://www.bls.gov/data/> |
| USDA Economic Research Service | Rural-urban classifications, poverty, food security | County | <https://www.ers.usda.gov/data-products/> |
| USDA Rural-Urban Continuum Codes | Classification of counties by urbanization | County | <https://www.ers.usda.gov/data-products/rural-urban-continuum-codes/> |

#### B.3 Health Behaviors and Risk Factors

| **Resource** | **Description** | **Geographic Level** | **URL** |
| --- | --- | --- | --- |
| BRFSS | Behavioral Risk Factor Surveillance System; smoking, obesity, physical activity, alcohol use | State (county estimates modeled) | <https://www.cdc.gov/brfss/> |
| CDC PLACES | Local health data for chronic disease, health behaviors, prevention | County, Census tract | <https://www.cdc.gov/places/> |
| County Health Rankings | Annual rankings of county health outcomes and health factors | County | <https://www.countyhealthrankings.org/> |
| National Health Interview Survey (NHIS) | Health behaviors, conditions, healthcare access | National, Regional | <https://www.cdc.gov/nchs/nhis/> |
| SAMHSA National Survey on Drug Use and Health | Substance use and mental health data | State, Regional | <https://www.samhsa.gov/data/data-we-collect/nsduh-national-survey-drug-use-and-health> |

#### B.4 Healthcare Access and System Data

| **Resource** | **Description** | **Geographic Level** | **URL** |
| --- | --- | --- | --- |
| Area Health Resources Files (AHRF) | Healthcare workforce, facilities, utilization | County | <https://data.hrsa.gov/topics/health-workforce/ahrf> |
| HRSA Data Warehouse | Health Professional Shortage Areas, Medically Underserved Areas | County, Service area | <https://data.hrsa.gov/> |
| CMS Geographic Variation Dashboard | Medicare spending, utilization, outcomes by region | County, HRR, State | <https://www.cms.gov/research-statistics-data-and-systems/statistics-trends-and-reports/medicare-geographic-variation> |
| Kaiser Family Foundation State Health Facts | Insurance coverage, Medicaid expansion, health policy data | State | <https://www.kff.org/statedata/> |
| Dartmouth Atlas of Health Care | Regional variations in medical care | HRR, State | <https://www.dartmouthatlas.org/> |
| AHRQ Healthcare Cost and Utilization Project (HCUP) | Hospital utilization data | State, National | <https://www.ahrq.gov/data/hcup/index.html> |

#### B.5 Environmental and Exposure Data

| **Resource** | **Description** | **Geographic Level** | **URL** |
| --- | --- | --- | --- |
| EPA Air Quality System (AQS) | Ambient air pollution monitoring data (PM2.5, ozone, etc.) | Monitor, County | <https://www.epa.gov/aqs> |
| EPA Environmental Justice Screening Tool (EJScreen) | Environmental and demographic indicators for EJ analysis | Census tract, County | <https://www.epa.gov/ejscreen> |
| EPA Toxics Release Inventory (TRI) | Industrial facility chemical releases | Facility, County | <https://www.epa.gov/toxics-release-inventory-tri-program> |
| CDC/ATSDR Social Vulnerability Index | Social vulnerability to environmental hazards | Census tract, County | <https://www.atsdr.cdc.gov/placeandhealth/svi/> |
| USGS Water Quality Data | Surface and groundwater quality monitoring | Site, Watershed | <https://www.usgs.gov/mission-areas/water-resources/science/water-quality> |
| Climate and Health Data Portal | Climate-related health impacts | County, State | <https://www.cdc.gov/climateandhealth/> |

#### B.6 Structural Racism and Segregation Measures

| **Resource** | **Description** | **Geographic Level** | **URL** |
| --- | --- | --- | --- |
| Diversity and Disparities Project | Residential segregation indices (dissimilarity, isolation, etc.) | MSA, County | <https://s4.ad.brown.edu/projects/diversity/> |
| National Community Reinvestment Coalition (NCRC) HOLC Maps | Digitized historical redlining maps | City, Neighborhood | <https://ncrc.org/holc/> |
| Mapping Inequality | University of Richmond digitized HOLC maps | City, Neighborhood | <https://dsl.richmond.edu/panorama/redlining/> |
| Opportunity Insights | Economic mobility data by race and geography | Census tract, County | <https://opportunityinsights.org/> |
| Urban Institute Data Tools | Segregation, housing, wealth inequality data | Various | <https://www.urban.org/data-tools> |
| Policy Link National Equity Atlas | Racial equity indicators | County, MSA, State | <https://nationalequityatlas.org/> |

#### B.7 Policy and Legislative Resources

| **Resource** | **Description** | **Geographic Level** | **URL** |
| --- | --- | --- | --- |
| Kaiser Family Foundation State Health Facts | Medicaid expansion, ACA enrollment, state health policy | State | <https://www.kff.org/statedata/> |
| NCSL State Legislation Database | State legislation on health, labor, environment | State | <https://www.ncsl.org/research.aspx> |
| Urban Institute State and Local Finance Initiative | State tax, spending, and fiscal policy data | State | <https://www.urban.org/policy-centers/cross-center-initiatives/state-and-local-finance-initiative> |
| Economic Policy Institute Minimum Wage Tracker | Current state and local minimum wage laws | State, Local | <https://www.epi.org/minimum-wage-tracker/> |
| Tax Policy Center State Tax Data | State tax policy comparisons | State | <https://www.taxpolicycenter.org/statistics/state-individual-income-tax-rates> |
| Guttmacher Institute State Policy Data | Reproductive health policy | State | <https://www.guttmacher.org/state-policy> |

#### B.8 International and Comparative Data

| **Resource** | **Description** | **Geographic Level** | **URL** |
| --- | --- | --- | --- |
| OECD Health Statistics | Comparative health data across OECD countries | National | <https://www.oecd.org/health/health-data.htm> |
| WHO Global Health Observatory | International mortality, life expectancy, health indicators | National, Regional | <https://www.who.int/data/gho> |
| GBD Compare (IHME) | Interactive global burden of disease visualization | National, Subnational (select countries) | <https://vizhub.healthdata.org/gbd-compare/> |
| Eurostat Regional Health Data | European subnational health data | NUTS2 regions | <https://ec.europa.eu/eurostat/web/health> |
| UN World Population Prospects | Global demographic and mortality projections | National | <https://population.un.org/wpp/> |
| Commonwealth Fund International Health Policy Surveys | Cross-national health system comparisons | National | <https://www.commonwealthfund.org/international-health-policy-center> |

#### B.9 Analytical Tools and Platforms

| **Resource** | **Description** | **Access** | **URL** |
| --- | --- | --- | --- |
| CDC WONDER Query System | Online query system for mortality and population data | Free, public | <https://wonder.cdc.gov/> |
| IPUMS NHGIS | Harmonized Census and ACS data with GIS boundary files | Free with registration | <https://www.nhgis.org/> |
| PolicyMap | Mapping platform with health, demographic, economic data | Subscription (free for government) | <https://www.policymap.com/> |
| SimplyAnalytics | Demographic and health data mapping | Subscription | <https://simplyanalytics.com/> |
| R (statistical software) | Open-source statistical computing | Free | <https://www.r-project.org/> |
| GeoDa | Open-source spatial analysis software | Free | <https://geodacenter.github.io/> |
| QGIS | Open-source geographic information system | Free | <https://qgis.org/> |

#### B.10 Congressional and Government Accountability Resources

| **Resource** | **Description** | **URL** |
| --- | --- | --- |
| Congressional Research Service (CRS) Reports | Nonpartisan policy analysis for Congress | <https://crsreports.congress.gov/> |
| Government Accountability Office (GAO) | Audits and evaluations of federal programs | <https://www.gao.gov/> |
| Congressional Budget Office (CBO) | Fiscal and economic analysis | <https://www.cbo.gov/> |
| Medicare Payment Advisory Commission (MedPAC) | Analysis of Medicare policy | <https://www.medpac.gov/> |
| Medicaid and CHIP Payment and Access Commission (MACPAC) | Analysis of Medicaid and CHIP policy | <https://www.macpac.gov/> |
| National Academies of Sciences, Engineering, and Medicine | Consensus studies on health policy | <https://www.nationalacademies.org/topics/health-and-medicine> |

#### B.11 Key Published Studies and Reports

The following landmark studies provide foundational evidence on geographic health disparities and are recommended for further reading:

| **Citation** | **Focus** | **Access** |
| --- | --- | --- |
| Chetty, R., et al. (2016). The association between income and life expectancy in the United States, 2001–2014. JAMA, 315(16), 1750–1766. | Income-longevity gradient | <https://doi.org/10.1001/jama.2016.4226> |
| Dwyer-Lindgren, L., et al. (2017). Inequalities in life expectancy among US counties, 1980 to 2014. JAMA Internal Medicine, 177(7), 1003–1011. | County-level trends | <https://doi.org/10.1001/jamainternmed.2017.0918> |
| Case, A., & Deaton, A. (2020). Deaths of Despair and the Future of Capitalism. Princeton University Press. | Mortality crisis in working-class America | <https://press.princeton.edu/books/hardcover/9780691190785> |
| Montez, J. K., et al. (2020). U.S. state policies, politics, and life expectancy. Milbank Quarterly, 98(3), 668–699. | State policy impacts | <https://doi.org/10.1111/1468-0009.12469> |
| Woolf, S. H., & Aron, L. (Eds.). (2013). U.S. Health in International Perspective: Shorter Lives, Poorer Health. National Academies Press. | International comparison | <https://doi.org/10.17226/13497> |
| Marmot, M., et al. (2020). Health Equity in England: The Marmot Review 10 Years On. Institute of Health Equity. | UK health inequalities | <https://www.health.org.uk/publications/reports/the-marmot-review-10-years-on> |
| National Academies. (2017). Communities in Action: Pathways to Health Equity. National Academies Press. | Framework for health equity | <https://doi.org/10.17226/24624> |
| Williams, D. R., & Collins, C. (2001). Racial residential segregation: A fundamental cause of racial disparities in health. Public Health Reports, 116(5), 404–416. | Segregation and health | <https://doi.org/10.1093/phr/116.5.404> |

#### B.12 Data Notes and Limitations

Researchers and policymakers utilizing these resources should note the following considerations:

1. **Temporal alignment:** Data sources have varying reference periods; users should ensure temporal consistency when combining datasets.
2. **Geographic concordance:** County boundaries and FIPS codes occasionally change; IPUMS NHGIS provides crosswalks for longitudinal analysis.
3. **Small-area estimation:** County-level behavioral data (e.g., from BRFSS) are modeled estimates with associated uncertainty; confidence intervals should be considered.
4. **Suppression:** Mortality data for small populations may be suppressed to protect confidentiality; alternative data sources or aggregation may be required.
5. **Ecological inference:** Associations observed at the county or state level cannot be directly attributed to individuals (ecological fallacy).
6. **Data currency:** Users should verify they are accessing the most recent data releases, as updates occur regularly.

For technical assistance with data access or analysis, Senate staff may contact the Congressional Research Service (CRS) or request GAO studies on specific topics.
